# Decreased dynamic variability of the cerebellum in the euthymic patients with bipolar disorder

**DOI:** 10.1186/s12888-024-05596-4

**Published:** 2024-02-19

**Authors:** Zhenzhu Chen, Zhifang Zhang, Feng Li, Lei Zhao, Qijing Bo, Yuan Zhou, Chuanyue Wang

**Affiliations:** 1 The National Clinical Research Center for Mental Disorders & Beijing Key Laboratory of Mental Disorders Beijing Anding Hospital, Capital Medical University, 100088 Beijing, China; 2 Beijing Institute for Brain Disorders Center of Schizophrenia, Beijing Anding Hospital, Capital Medical University, 100088 Beijing, China; 3 Advanced Innovation Center for Human Brain Protection, Capital Medical University, 100069 Beijing, China; 4 CAS Key Laboratory of Behavioral Science, Institute of Psychology, 100101 Beijing, China; 5 Department of Psychology, University of Chinese Academy of Sciences, 100049 Beijing, China

**Keywords:** Bipolar disorder, Resting-state fMRI, Dynamic fractional amplitude of low-frequency fluctuations, Dynamic degree centrality, Cerebellum posterior lobe

## Abstract

**Background:**

Bipolar disorder (BD) is a complex mental illness characterized by different mood states, including depression, mania/hypomania, and euthymia. This study aimed to comprehensively evaluate dynamic changes in intrinsic brain activity by using dynamic fractional amplitude of low-frequency fluctuations (dfALFF) and dynamic degree centrality (dDC) in patients with BD euthymia or depression and healthy individuals.

**Methods:**

The resting-state functional magnetic resonance imaging data were analyzed from 37 euthymic and 28 depressed patients with BD, as well as 85 healthy individuals. Using the sliding-window method, the dfALFF and dDC were calculated for each participant. These values were compared between the 3 groups using one-way analysis of variance (ANOVA). Additional analyses were conducted using different window lengths, step width, and window type to ensure the reliability of the results.

**Results:**

The euthymic group showed significantly lower dfALFF and dDC values of the left and right cerebellum posterior lobe compared with the depressed and control groups (cluster level *P*_FWE_ < 0.05), while the latter two groups were comparable. Brain regions showing significant group differences in the dfALFF analysis overlapped with those with significant differences in the dDC analysis. These results were consistent across different window lengths, step width, and window type.

**Conclusions:**

These findings suggested that patients with euthymic BD exhibit less flexibility of temporal functional activities in the cerebellum posterior lobes compared to either depressed patients or healthy individuals. These results could contribute to the development of neuropathological models of BD, ultimately leading to improved diagnosis and treatment of this complex illness.

**Supplementary Information:**

The online version contains supplementary material available at 10.1186/s12888-024-05596-4.

## Highlights

This study highlights the importance of investigating dynamic intrinsic brain activity in patients with bipolar disorder (BD) during different mood states.

The patients with euthymic BD showed significantly less flexibility of temporal functional activities in the left and right cerebellum posterior lobe compared to patients with depressed BD or healthy individuals.

The use of dynamic fractional amplitude of low-frequency fluctuations and dynamic degree centrality allowed for a comprehensive analysis of brain activity, providing reliable and objective results.

**Supplementary Information:**

The online version contains supplementary material available at 10.1186/s12888-024-05596-4.

## Introduction

Bipolar disorder (BD) is a serious mental illness characterized by alternating mood states of depression, mania/hypomania and euthymia. These changes adversely affect emotion, cognition, activity level, social function, and quality of life even when the patient is euthymic [1]. The diversity of mood states, which occur over the entire course of BD, greatly challenges clinical decisions regarding diagnosis and treatment. Before the disease is fully exposed BD may be misdiagnosed as unipolar depression, and in error patients are easily given antidepressant monotherapy [2]. On the other hand, hypomania is often ignored by the patient and those around them, or sometimes the manic state is difficult to distinguish from schizophrenia [3]. Clinical characteristics associated with different mood states may reflect specific pathological alterations [4], including cognitive and functional impairment [5, 6]. Therefore, it is of great importance to differentiate the mood states of BD, including the euthymic.

Resting-state functional magnetic resonance imaging (fMRI) is a technique that measures the intrinsic, spontaneous activity of the brain during rest, which consumes a significant amount of energy [7]. This makes resting-state fMRI a valuable tool for studying the underlying neurobiological mechanisms of neuropsychiatric diseases, including BD [8]. Resting-state fMRI provides various functional metrics that can supply disease-related information from different perspectives. For example, the fractional amplitude of low-frequency fluctuations (fALFF) is a functional metric that reflects the intensity of spontaneous brain activity from a given brain region. fALFF is derived from the amplitude of low-frequency fluctuations (ALFF) and represents the relative contribution of low frequency fluctuations within the frequency range. The advantages of fALFF as an evaluative functional metric include eliminating the influence of physiological noise and its better sensitivity and specificity for detecting spontaneous brain activity [9]. Degree centrality (DC) is another functional metric provided by resting-state fMRI. It is a derivative indicator based on functional connectivity and graph theory, which can reflect the importance of specific nodes in the brain functional connectome [10]. By measuring the degree of connectivity between different brain regions, DC can provide valuable information about the underlying neural networks that support cognitive and behavioral processes. Overall, the functional metrics provided by resting-state fMRI, such as fALFF and DC, can provide valuable insights into the underlying neurobiological mechanisms of neuropsychiatric diseases and better understand the complex functioning of the brain in health and disease.

Several studies have investigated the fALFF and DC in patients with BD. Compared to healthy individuals, patients with depressed BD have shown lower fALFF values in the left cerebellum posterior lobe (CPL) [11], right middle temporal gyrus [12] and right lingual gyrus [13]. However, they have shown higher fALFF values in the right inferior temporal gyrus [12] and bilateral superior frontal gyrus [13]. Patients with euthymic BD have shown higher fALFF values in the left superior temporal gyrus, superior frontal gyrus, middle occipital gyrus, bilateral thalamus and right putamen [14]. Regarding DC as an index, a study by Russo et al. [4] reported that patients with depressed bipolar type 1 demonstrated a deficit of the sensorimotor network. The findings suggest that patients with BD in different mood states experience differences in pathological alterations, as revealed by fALFF and DC analyes.

Recent studies have recognized that the intrinsic activity of brain regions and brain networks are temporally variable and dynamic [15]. However, in all the studies described above, both the fALFF and DC values were calculated as static indices across the scanning time. Therefore, the dynamics of spontaneous brain activity during scanning have not been fully elucidated, despite the likelihood that the variability and flexibility of brain activity of specific regions and the entire neuronal network are important [16].

The investigation of dynamic fMRI metrics in BD is at an early stage. To the best of our knowledge, there has been only one study of dynamic fALFF (dfALFF) in patients with BD [17]. In this study, individuals with depressed BD had significantly lower dfALFF variability in the right middle and left inferior temporal gyrus compared to the healthy individuals. No study has investigated the dynamic DC (dDC) values in patients with BD and there are no studies that compare the dynamic spontaneous brain activity in patients with BD in different mood states.

When collecting fMRI data in patients with BD, it is easier to obtain data from patient with euthymic or depressed BD compared with to those who are manic. As a result, in previous studies, most patients have been either euthymic or depressed. According to Judd et al., the percentages of the BD population that are depressed, euthymic, and manic are 53%, 31%, and 16% respectively [18]. Since the majority of the BD population (84%) is non-manic, studies targeted at either euthymic or depressed patients may reveal the brain function alterations during most of the disease course.

The present study aims to investigate dynamic dysfunction during non-manic states of BD. The resting-state fMRI data were collected from our previous study [19], including depressed or euthymic populations as well as a healthy individual group. The sliding-window method was applied to calculate the dfALFF values and dDC values for each subject, and these values were compared among the three subject groups in a voxel-wise way. To validate the results, data analyses were repeated with different window lengths, step width, and window type. This type of analysis of dynamic functional metrics from a whole-brain view ensures that the method is data-driven and objective. The findings of this study will shed light on the dynamic dysfunction of specific brain regions during non-manic states of BD, providing valuable information for understanding BD pathology and developing neurobiological models of BD.

## Methods

The Institutional Review Board of the Brain Image Center, Beijing Normal University and Beijing Anding Hospital, Capital Medical University approved this study. All participants provided prior signed consent.

### Subjects

The initial data included that of 65 patients with BD and 85 healthy individuals, collected during our previous study, with the same inclusion and exclusion criteria and patients’ medication use [19]. In brief, the patients were all from Beijing Anding Hospital, Capital Medical University. Diagnoses of BD were based on the Structured Clinical Interview for DSM-IV Axis I Disorder-Patient Edition (SCID-I/P) [20]. The SCID assessments in this study were conducted by trained psychiatrists who possess extensive experience and expertise in diagnosing and assessing mental health conditions. Patients with bipolar disorder (BD) who had a history of comorbid psychiatric disorders were excluded from the study. Patients’ depressive symptoms were assessed with the 17-item Hamilton Depression Rating Scale (HAMD) [21], and manic symptoms using the Young Mania Rating Scale (YMRS) [22]. Patients with YMRS scores ≤ 6 were included. Patients with BD and HAMD scores ≤ 7 were regarded as euthymic. Healthy individuals for the control group were recruited from nearby communities and screened using the non-patient version of the SCID. The handedness was assessed using the Chinese revised version of the Edinburgh handedness inventory [23]. And all the participants were right-handed, Han Chinese, with a primary or higher education. Because of large head motion (see our previous study [19]), 7 patients with euthymic BD, 2 patients with depressed BD, and 14 healthy individuals were excluded.

Finally, the study population comprised 56 patients (30 euthymic and 26 depressed) and a control group of 70 healthy individuals (Table 1).

**Table 1:** Demographic and clinical factors of the patients with BD and the healthy controls

					Post-hoc, T (P_Bonferroni_) ^a^		
	EP	DP	HC	F/χ2 (*P*)	EP cf. HC	DP cf. HC	DP cf. EP
Age ^b^	27.20 ± 8.34	32.19 ± 13.01	30.61 ± 10.87	1.64 (0.198)	—	—	—
Gender (male/female) ^c^	21/9	14/12	34/37	4.16 (0.125)	—	—	—
Education ^c,d^	5/10/7/8	2/12/5/7	6/15/18/32	9.06 (0.170)	—	—	—
FD ^b^	0.15 ± 0.06	0.14 ± 0.04	0.13 ± 0.04	1.05 (0.352)	—	—	—
HAMD ^e^	2.27 ± 2.02	15.58 ± 6.22	0.75 ± 1.52	207.44 (< 0.001)	4.10 (< 0.001)	18.30 (< 0.001)	11.09 (< 0.001)

Abbreviations: DP, patients with depressed BD; EP, Patients with euthymic BD; HC, healthy controls; FD, framewise displacement; HAMD, Hamilton depression scale

^a^ Post-hoc analysis was conducted by using two-sample t test and bonferroni correction;

^b^ These variables were compared using one-way ANOVA;

^c^ These variables were compared using χ2 tests;

^d^ Education = Junior high school / Senior high school or special secondary school / Junior college / Bachelor degree or above;

^e^ This variable was compared using Kruskal-Wallis H test

### Imaging data acquisition

A Siemens TIM Trio 3T system (Siemens, Erlangen, Germany) was used to scan the subjects at the Brain Imaging Center of Beijing Normal University. T1 images (128 volumes) were acquired after resting-state fMRI scanning (240 volumes). During the resting state, the subjects were required to keep their eyes closed, stay awake, and keep their body still. Functional images were collected using the Echo-Planar Imaging (EPI) sequence: 33 axially slices, interlaced scanning, repetition time (TR) 2000 ms, echo time (TE) 30 ms, flip angle (FA) 90°, field of view (FOV) 200 × 200 mm^2^, matrix size 64 × 64, slice thickness 3.5 mm, and voxel size 3.13 × 3.13 × 4.2 mm^3^. T1 images were collected with the T1-weighted 3D magnetization-prepared rapid gradient echo (MPRAGE) sequence: sagittal collected, TR 2530 ms, TE 3.39 ms, FA 7°, FOV 256 × 192 mm^2^, matrix size 192 × 192, thickness 1.33 mm, and voxel size 1 × 1 × 1.33 mm^3^.

### Resting-state fMRI data preprocessing

All preprocessed steps were performed in DPABI v5.1 (http://rfmri.org/DPABI) [24]. The preprocessing steps consisted of the following: the first 5 timepoints were discarded; slice timing correction; realign; regression of several covariates (i.e., Friston-24 parameters of head motion, head motion scrubbing regressor, signals of the white matter and cerebrospinal fluid); normalization (T1 images and DARTEL toolbox, voxel size = 3 × 3 × 3 mm^3^); detrending; and band-pass filtering (0.01–0.1 Hz for DC analysis only). The framewise displacement (FD) and mean FD values were calculated as in our previous study [19].

### dfALFF and dDC analyses

dfALFF and dDC were calculated by the sliding-window method [25, 26] in DPABI v5.1. In these calculations, the Temporal Dynamic Analysis toolkits were used. Referring to one recent study [16], 32 TRs window length, 2 TRs step width, and the Hamming sliding window were selected for the dfALFF and dDC analyses. Thus, 102 sliding windows were obtained. For each sliding window, fALFF and DC values were calculated separately via a voxel-wise method. The fALFF was calculated using the signal strength of the low frequency range (i.e., 0.01–0.1 Hz) to divide the detectable entire frequency range [9]. The DC value was the sum of the Pearson’s correlation coefficients of all possible pairs of voxels (correlation threshold of r_0_ was set at 0.25) [10, 27].

The fALFF and DC maps for each participant were converted into z-score maps using Fisher’s r-to-z transformation. The standard deviation of z-value maps across the 102 sliding-window was used to perform the dynamic maps. dfALFF maps and dDC maps were computed respectively. Finally, z-standardization was applied for the dfALFF maps and dDC maps. These dynamic maps were smoothed using a 4-mm full-width at half-maximum Gaussian kernel. The smoothed dynamic maps were entered into the second-level analyses.

### Statistical analysis

Calculations of differences in demographic and clinical factors were conducted with SPSS 20.0 (SPSS, IL, USA) software. One-way analysis of variance (ANOVA) and post-hoc analyses were employed to compare differences in demographic factors across the euthymic BD, depressed BD, and control groups. The chi-squared test was used to compare gender ratios and educational levels. Regarding the HAMD scores, the Shapiro-Wilk test revealed that the HAMD scores of the three groups did not follow a normal distribution. Therefore, the Kruskal-Wallis H test was employed to compare the HAMD scores across the three groups. In cases where a significant difference in HAMD scores was observed among the groups, post hoc T-test and Bonferroni correction (*P*_Bonferroni_ = *P*_uncorrected_× 3 < 0.05) was applied to compare pairs of groups.

The second-level analysis for dfALFF was conducted in SPM12 (Wellcome Department of Cognitive Neurology, London, UK). One-way ANOVA was applied to compare differences across the euthymic BD, depressed BD, and control groups, with age, gender, education level, and mean FD as nuisance covariates. Multiple comparisons correction was performed via the cluster-level Family Wise Error (FWE) correction. Cluster-level threshold was set at *P*_FWE_ < 0.05 and voxel-level threshold at *P*_uncorrected_ < 0.001. For the regions surviving the threshold, post-hoc two sample T-test and Bonferroni correction were conducted to compare pairs of groups (*P*_Bonferroni_ = *P*_uncorrected_× 3 < 0.05). The same second-level analysis method was used for the dDC analysis. In the first-level and second-level dfALFF and dDC analyses, a gray matter mask was employed to limit the scope of our data analysis. The gray matter mask was made similarly with the method described in our previous study [19].

Within the HC group, EP group, DP group, and the combined group, potential associations were examined between HAMD scores and left dynamic ALFF, right dynamic ALFF, left dynamic DC, and right dynamic DC (brain regions with inter-group differences) using Pearson correlation analyses. A statistically significant threshold was set at *P*_Bonferroni_ = *P*_uncorrected_× 4 < 0.05.

### Validation analyses

To verify our main results when 32 TRs/2 TRs/hamming was applied, other window lengths (16 TRs, 24 TRs, 48 TRs and 64 TRs), step width (1 TR) and window type (rectwin sliding window) were used to re-calculate the dfALFF and dDC values. The second-level analyses were conducted as described for the statistical analyses (above).

## Results

### Demographic and clinical characteristics

Among the 3 groups, the subjects were comparable regarding all demographic characteristics (*P*-values > 0.05). There were statistically significant differences in HAMD scores among the three groups (F = 207.44, *P* < 0.001). And the further post-hoc analysis revealed that the patients with depressed BD had significantly higher HAMD scores compared with the other two groups (DP vs. HC: T = 18.30, *P*_Bonferroni_ < 0.001; DP vs. EP: T = 11.09, *P*_Bonferroni_ < 0.001), as indicated in Table 1. The healthy control group showed the lowest HAMD scores.

### dfALFF and dDC

**Fig. 1:**
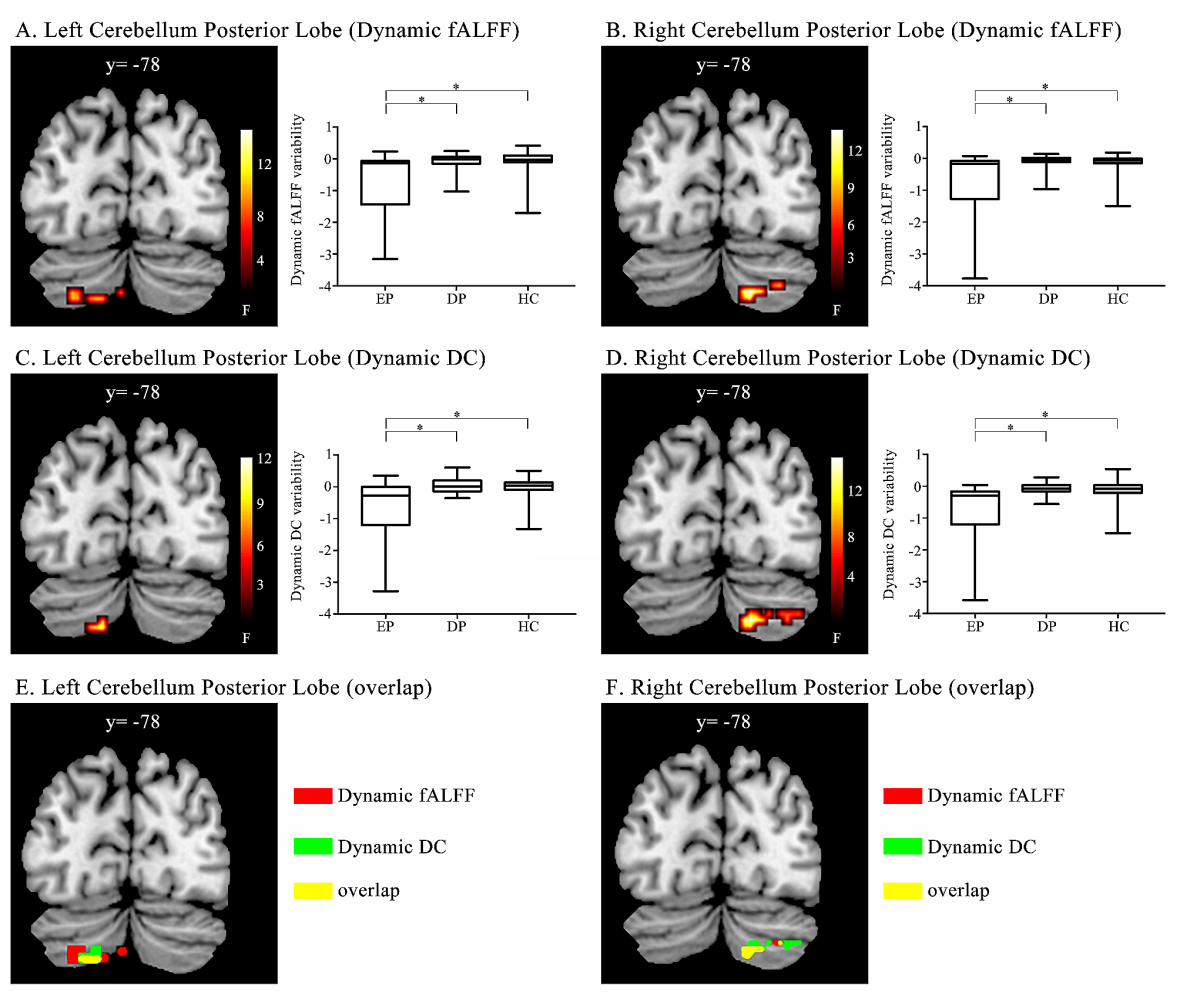
Significant differences were found between the patients with euthymic BD and patients with depressed BD, and between patients with euthymic BD and healthy individuals (32TR/2step/hamming). **(A and B)** The left **(A)** and right **(B)** brain region revealed by the dfALFF analysis. **(C and D)** The left **(C)** and right **(D)** brain region revealed by the dDC analysis. **(E and F)** The overlapped brain regions found in the **(E)** dfALFF and **(F)** dDC analysis

Based on the one-way ANOVA, the dfALFF and dDC values of the 3 groups each differed significantly, in the left and right CPL. Table 2 displays the mean, variance, and median of the dynamic fALFF and dynamic DC in the CPL for three groups: the depressed BD patients, the healthy controls, and the euthymic BD patients. The post-hoc analysis showed that the fALFF values of the euthymic BD group were significantly lower than that of either of the other groups (Fig. 1A and B, and Table 3), while the depressed BD and control groups were comparable (*P*_Bonferroni_ > 0.05). Similarly, in the post-hoc analysis, the dDC values of the euthymic BD group were significantly lower than that of either of the other 2 groups (Fig. 1C and D, and Table 3), and the depressed BD and controls groups did not differ significantly (*P*_Bonferroni_ > 0.05).

**Table 2:** The mean, variance and median of the dynamic fALFF and dynamic DC in the CPL for three groups: the depressed BD patients, the healthy controls, and the euthymic BD patients (32TR/2-step/hamming)

	Hemisphere	Group	Mean ± Variance	Median
Dynamic ALFF	Left	DP	-0.043 ± 0.014	-0.044
		HC	-0.070 ± 0.120	-0.034
		EP	-0.742 ± 1.059	-0.142
	Right	DP	-0.104 ± 0.019	-0.068
		HC	-0.109 ± 0.067	-0.067
		EP	-0.748 ± 1.473	-0.126
Dynamic DC	Left	DP	0.005 ± 0.067	0.018
		HC	-0.030 ± 0.088	0.030
		EP	-0.625 ± 0.930	-0.237
	Right	DP	-0.120 ± 0.037	-0.093
		HC	-0.106 ± 0.076	-0.080
		EP	-0.754 ± 1.281	-0.215

Abbreviations: DP, patients with depressed BD; EP, patients with euthymic BD; HC, healthy controls

**Table 3:** Results of the dynamic fALFF and dynamic DC analyses of the CPL among the euthymic BD patients, the depressed BD patients, and the healthy controls (32TR/2-step/hamming ^a^), EP cf. DP cf. HC

						Post–hoc analysis	
	Hemisphere	MNI coordinates	Peak F/T	Cluster size	Cluster-level, *P*_FWE_	Comparisons	*P*_Bonferroni_
Dynamic ALFF	Left	–3, − 75, − 45	14.75	23	0.010	EP < DP	< 0.001*
						EP < HC	< 0.001*
	Right	15, − 78, − 42	13.61	54	< 0.001	EP < DP	< 0.001*
						EP < HC	< 0.001*
Dynamic DC	Left	–6, − 72, − 45	12.13	26	0.04	EP < DP	< 0.001*
						EP < HC	< 0.001*
	Right	15, − 78, − 42	15.38	79	< 0.001	EP < DP	< 0.001*
						EP < HC	< 0.001*

Abbreviations: DP, patients with depressed BD; EP, patients with euthymic BD; HC, healthy controls

*P < 0.05

The regions of the left and right CPL showing alterations, indicated by the dfALFF and dDC analyses, overlapped. Specifically, there were 9 and 42 overlapped voxels in the left and right CPL, respectively (Fig. 1E and F).

The correlation analyses indicated that there was a significant positive correlation between HAMD score and right dynamic DC in the DP group (*r* = 0.571, *P*_Bonferroni_ = 0.008).

### Validation analyses

The dfALFF and dDC analyses were repeated with other window lengths (16, 24, 48, or 64 TRs), step width (1 TR), and window type (rectwin sliding window). When window size/window step/window type were set to 16 TRs/2 TRs/hamming, 24 TRs/2 TRs/hamming, or 32 TRs/2 TRs/rectwin, significant differences in dynamic fALFF and dynamic DC values were each observed in the left and right CPLs, and areas of significance in dynamic fALFF and dynamic DC analyses overlapped (Table 4, and supplementary materials). Under the 48 TRs/2 TRs/hamming, and 64 TRs/2 TRs/hamming, significant differences in dynamic fALFF and dynamic DC values were observed only in the right CPL, and areas of significance overlapped (Table 4, and supplementary materials). Applying 32 TRs/1 TRs/hamming, significant differences in dynamic fALFF values were observed in the left and right CPLs; significant differences in dynamic DC values were only located in the right CPL, and areas of significance in dynamic fALFF and dynamic DC analyses overlapped (Table 4, and supplementary materials).

**Table 4:** Dynamic fALFF and dynamic DC analyses of the CPL among the euthymic BD patients, the depressed BD patients, and the healthy controls, by different window size/window step/window type

							Post–hoc analysis	
	Analysis	Hemisphere	MNI coordinates	Peak F values	Cluster size	Cluster-level *P*_FWE_	Comparisons	*P*_Bonferroni_
16TR/2-step/hamming	Dynamic fALFF	Left	–3, − 75, − 45	13.80	38	< 0.001*	EP < DP	< 0.001*
							EP < HC	< 0.001*
		Right	15, − 78, − 42	14.01	57	< 0.001*	EP < DP	< 0.001*
							EP < HC	< 0.001*
	Dynamic DC	Left	–6, − 75, − 45	14.18	46	0.001*	EP < DP	< 0.001*
							EP < HC	< 0.001*
		Right	15, − 78, − 42	14.08	81	< 0.001*	EP < DP	< 0.001*
							EP < HC	< 0.001*
24TR/2-step/hamming	Dynamic fALFF	Left	–3, − 75, − 45	14.98	31	0.001*	EP < DP	< 0.001*
							EP < HC	< 0.001*
		Right	15, − 78, − 42	14.23	62	< 0.001*	EP < DP	< 0.001*
							EP < HC	< 0.001*
	Dynamic DC	Left	–6, − 75, − 45	13.06	34	0.008*	EP < DP	< 0.001*
							EP < HC	< 0.001*
		Right	15, − 78, − 42	14.20	81	< 0.001*	EP < DP	< 0.001*
							EP < HC	< 0.001*
48TR/2-step/hamming	Dynamic fALFF	Right	18, − 78, − 42	11.98	47	< 0.001*	EP < DP	< 0.001*
							EP < HC	< 0.001*
	Dynamic DC	Right	15, − 78, − 42	15.08	66	< 0.001*	EP < DP	< 0.001*
							EP < HC	< 0.001*
64TR/2-step/hamming	Dynamic fALFF	Right	21, − 78, − 42	11.34	35	0.001*	EP < DP	< 0.001*
							EP < HC	< 0.001*
	Dynamic DC	Right	24, − 72, − 42	12.93	55	< 0.001*	EP < DP	< 0.001*
							EP < HC	< 0.001*
32TR/2-step/rectwin	Dynamic fALFF	Left	–3, − 75, − 45	13.83	20	0.025*	EP < DP	< 0.001*
							EP < HC	< 0.001*
		Right	18, − 78, − 42	12.11	57	< 0.001*	EP < DP	< 0.001*
							EP < HC	< 0.001*
	Dynamic DC	Right	21, − 75, − 45	11.69	62	< 0.001*	EP < DP	< 0.001*
							EP < HC	< 0.001*
32TR/1-step/hamming	Dynamic fALFF	Left	–3, − 75, − 45	14.71	23	0.010*	EP < DP	< 0.001*
							EP < HC	< 0.001*
		Right	15, − 78, − 42	13.59	54	< 0.001*	EP < DP	< 0.001*
							EP < HC	< 0.001*
	Dynamic DC	Left	–6, − 72, − 45	12.10	26	0.040*	EP < DP	< 0.001*
							EP < HC	< 0.001*
		Right	15, − 78, − 42	15.29	81	< 0.001*	EP < DP	< 0.001*
							EP < HC	< 0.001*

Abbreviations: DP, patients with depressed BD; EP, patients with euthymic BD; HC, healthy controls

^*^P < 0.05

## Discussion

This study investigated the dynamic spontaneous brain activity in non-manic states of BD by using dfALFF and dDC. The resting-state fMRI data of 37 euthymic and 28 depressed patients with BD as well as 85 healthy individuals were analyzed using the sliding-window method. The results showed that both the dfALFF values and the dDC values were lower in the left and right CPL of patients with euthymic BD, compared to patients with depressed BD or the healthy control group. The findings were validated by repeating the analysis with different window lengths, step width, and window type.

These results led us to focus attention on the cerebellum and its subregions. Anatomically, the cerebellum of the normal human is divided into 10 lobules, I to X. These can be further classified as the anterior lobe (lobules I-V), posterior lobe (lobules VI-IX, including Crus I and Crus II and lobule VIIb), and flocculonodular lobe (lobule X) [28, 29]. Several studies have suggested that the CPLs are involved in emotion regulation [30, 31], working memory [32] and executive function [33]. The posterior lobe of the cerebellum is reportedly activated during responses to all 5 primary emotions (sadness, happiness, anger, fear and disgust) [34]. In another study, functional neuroimaging data of the subjects as they viewed a dramatic film indicated dynamic perceptual and affective processes in regions of the posterior and inferior cerebellum, and a dynamic interaction with higher order regions in the cerebral cortex [35]. In patients with cerebellum stroke, lesions in posterior lobe regions led to deficits in language, visual spatial, and executive functions, and affective dysregulation [36]. These findings in healthy individuals and patients with cerebellum lesions consistently indicate that the CPL is involved in higher brain functions, especially dysfunctional emotion, which is a characteristic of BD [37, 38].

Previous structural and functional neuroimaging studies have suggested that the cerebellum is involved in the pathophysiology of BD. Several studies have reported abnormal cerebellar structure in patients with BD, including reduced gray matter volume [39, 40] and abnormal microstructure [41]. In addition, several task-related fMRI studies have revealed brain dysfunction across different mood states of BD. For example, relative to healthy individuals, patients with euthymic BD demonstrated lower sensitivity to pain and significantly lower brain activity in the left cerebellum when experiencing pain [42]. During a task contrasting emotional with neutral distractors, pediatric patients with BD showed higher CPL activity compared with healthy individuals [43]. A meaningful case report showed that the subject developed a manic mood state with the incidence of a cerebellum sub-region lesion that involved the left lobules VI, VIIa (crus I), and IX, and the posterior area of the vermis [44]. Consistent with the above, in the present study, the dynamic functional activities in the left and right CPL of patients with euthymic BD were lower relative to that of either the patients with depressed BD or the control group. Altogether, these findings may provide evidence that the CPL is of crucial importance in the pathophysiology of BD.

In this study, the lower dfALFF values implied that the patients with euthymic BD had lower temporal variability and flexibility of regional brain activity in the left and right CPLs compared with healthy individuals. The dfALFF values of the patients with depressed BD were similar to that of the controls. No previous studies have conducted dfALFF analysis in patients with euthymic BD.

Recently, Sun et al. [17] reported that patients with depressed BD I showed lower dfALFF temporal variability in the right middle temporal gyrus and left inferior temporal gyrus, compared with healthy individuals. This contradicts the negative results of the present study regarding depressed BD patients. The discrepancies may be related to different sample size (65 herein cf. 40) and the heterogeneity of BD subjects: the present study included both BD I and BD II patients, but the population in Sun et al. [17] was entirely BD I.

In another study, Yu et al. [11] demonstrated lower static fALFF values in the left CPL in patients with depressed BD. We speculate that the lower dynamic variability of spontaneous brain activity in the CPLs of patients with euthymic BD may reflect a compensatory effect to maintain a relatively normal mood state. Therefore, lower dfALFF values may be a potential biomarker of euthymic mood state in BD.

Unlike fALFF, which can measure the local brain activity of specific brain regions, DC measures the centrality of specific brain regions from the perspective of the brain connectome. In the present study, regions revealed in the dDC analysis overlapped with those of the dfALFF analysis. The patients with euthymic BD showed lesser dynamic variability and flexibility of centrality of integrating global resting-state activity in the left and right CPLs compared with the depressed BD group or control, while the latter two groups were similar. To our best knowledge, no previous studies have conducted dDC analyses in patients with euthymic BD and depressed BD.

DC is an analysis index based on graph theory. Other indices using the graph theory approach have also found abnormalities in the CPL of patients with BD. For example, the nodal characteristics (nodal efficiency and nodal strength) of the left CPL were lower in unmedicated patients with depressed BD [45]. Whole-brain connectivity analyses suggested that patients with euthymic BD had significantly lower functional connectivity strength in the bilateral CPLs [46]. Within-network analysis revealed lower connectivity in the cerebellum resting-state networks in patients with euthymic BD [47]. Although our study did not include patients with manic BD, such patients have also been found with abnormal static DC in the CPL [48]. As we speculated on the causes of abnormal dfALFF values in the patients with euthymic BD (above), the lower dDC values shown by the patients in this group may be a compensatory effect to keep the mood relatively stable, making it another potential biomarker of euthymic mood state in BD. Additionally, it is evident that the EP group exhibits high variance in both dfALFF and dDC analyses. This suggests that the extent of dfALFF and dDC reduction needed to maintain emotional stability varies greatly among individuals within the EP group. Some EP patients may require a lesser degree of dfALFF and dDC reduction, while others may require a greater degree. The correlation analysis confirms that the higher variability of dynamic DC in the right cerebellum posterior lobe (indicating lower stability) is associated with more severe depressive symptoms.

This study has several limitations that should be acknowledged. Firstly, the sample size is relatively small and thus the present results should be interpreted with caution. Future studies with larger sample size are necessary to validate these findings. Secondly, most of the patients in this study had received various psychotropic medications, which may have confounded the analysis of analyzing dynamic brain activity. Recruiting unmedicated individuals in the future may provide more reliable results. Thirdly, BD patients were categorized into depressed and euthymic groups based on their current emotional state, without taking into account the specific subtypes of BD. Further research can be conducted to explore the influence of different subtypes on the results. Fourthly, it is important to acknowledge that certain clinical factors such as illness duration, history of psychosis during previous episodes, age of onset of the disease, etc., may act as confounders in the research findings. We will consider these factors in future studies to obtain a more comprehensive understanding of their impact. Finally, data regarding the number of mood states that the patients had experienced was not collected. Since the number and predominant polarity of mood states can guide clinical medication and predict outcomes [49, 50], and may also affect dynamic brain function. Therefore, future studies should consider collecting this information to better understand the relationship between mood states and dynamic brain activity in patients with BD.

## Conclusion

This study found that the dDC and dfALFF values of the left and right CPLs of patients with euthymic BD were lower than that of patients with depressed BD or healthy individuals, while the values of the latter two groups were similar. These findings provide further evidence for the possible role of functional alterations in the cerebellum in the pathology of BD, and could contribute to the development of neuropathological models of this disorder.

### Electronic supplementary material

Below is the link to the electronic supplementary material.

Supplementary Material 1

## Data Availability

The datasets used and/or analysed during the current study are available from the corresponding author on reasonable request.
