# Supplementary material for: Decreased dynamic variability of the cerebellum in the euthymic patients with bipolar disorder
Source: BMC Psychiatry. 2024 Feb 19;24:137. doi: 10.1186/s12888-024-05596-4 (PMC10877821; doi:10.1186/s12888-024-05596-4)

**Supplementary Materials**

**RESULTS**

The post-hoc analyses (below) all showed that the euthymic BD patients exhibited lower dynamic fALFF and dynamic DC values relative to either the depressed BD patients or the healthy control group. The dynamic fALFF and dynamic DC values of the depressed BD patients and the healthy controls were statistically comparable.

**Window size/window step/window type: 16 TRs/2 TRs/hamming**

Significant differences in dynamic fALFF and dynamic DC values were observed in the left and right cerebellum posterior lobes (Figure S1), and areas of significance overlapped. The number of overlapped voxels was 29 (Figure S1E) and 39 (Figure S1F).

**Window size/window step/window type: 24 TRs/2 TRs/hamming**

Significant differences in dynamic fALFF and dynamic DC values were observed in the left and right cerebellum posterior lobes (Figure S2), and areas of significance overlapped. The number of overlapped voxels was 15 (Figure S2E) and 44 (Figure S2F).

**Window size/window step/window type: 48 TRs/2 TRs/hamming**

Significant differences in dynamic fALFF and dynamic DC values were observed only in the right cerebellum posterior lobe (Figure S3), and areas of significance overlapped. The number of overlapped voxels was 39 (Figure S3C).

**Window size/window step/window type: 64 TRs/2 TRs/hamming**

Significant differences in dynamic fALFF and dynamic DC values were observed only in the right cerebellum posterior lobe (Figure S4), and areas of significance overlapped. The number of overlapped voxels was 27 (Figure S4C).

**Window size/window step/window type: 32 TRs/2 TRs/rectwin**

Significant differences in dynamic fALFF values were observed in the left and right cerebellum posterior lobes; significant differences in dynamic DC values were only located in the right cerebellum posterior lobe (Figure S5). Areas of significance in dynamic fALFF and dynamic DC analyses overlapped. The number of overlapped voxels was 38 (Figure S5D).

**Window size/window step/window type set at 32 TRs/1 TRs/hamming**

Significant differences in dynamic fALFF and dynamic DC values were observed in the left and right cerebellum posterior lobes (Figure S6), and areas of significance overlapped. The number of overlapped voxels was 9 (Figure S6E) and 42 (Figure S6F).

**Legends for supplementary figures**

**Figure S1.** Repeated analysis with 16TR/2step/hamming. (A, B) Dynamic fALFF. (A) Significant brain region of interest. (B) Averaged dynamic fALFF values within each group. (C, D) Dynamic DC analysis. (C) Significant brain region of interest. (D) Averaged dynamic DC values within each group. (E, F) Overlap of brain regions of interest in the (E) dynamic fALFF and (F) dynamic DC analyses.

**Figure S2.** Repeated analysis with 24TR/2step/hamming. (A, B) Dynamic fALFF.

(A) Significant brain region of interest. (B) Averaged dynamic fALFF values within each group.

(C, D) Dynamic DC analysis. (C) Significant brain region of interest. (D) Averaged dynamic DC values within each group. (E, F) Overlap of brain regions of interest in the (E) dynamic fALFF and (F) dynamic DC analyses.

**Figure S3.** Repeated analysis with 48TR/2step/hamming. (A, B) Dynamic fALFF. (A) Significant brain region of interest. (B) Averaged dynamic fALFF values within each group. (C) Overlap of brain regions of interest in the dynamic fALFF and dynamic DC analyses.

**Figure S4.** Repeated analysis with 64TR/2step/hamming. (A, B) Dynamic fALFF. (A) Significant brain region of interest. (B) Averaged dynamic fALFF values within each group. (C) Overlap of brain regions of interest in the dynamic fALFF and dynamic DC analyses.

**Figure S5.** Repeated analysis with 32TR/2step/rectwin. (A, B) Dynamic fALFF. (A) Significant brain region of interest. (B) Averaged dynamic fALFF values within each group. (C) Left: the significant brain region found in the dynamic DC analysis. Right: averaged dynamic DC values within each group. (D) Overlap of brain regions of interest in the dynamic fALFF and dynamic DC analyses.

**Figure S6.** Repeated analysis with 32TR/1step/hamming. (A, B) Dynamic fALFF. (A) Significant brain region of interest. (B) Averaged dynamic fALFF values within each group. (C, D) Dynamic DC analysis. (C) Significant brain region of interest. (D) Averaged dynamic DC values within each group. (E, F) Overlap of brain regions of interest in the (E) dynamic fALFF and (F) dynamic DC analyses.

**Figure S1**


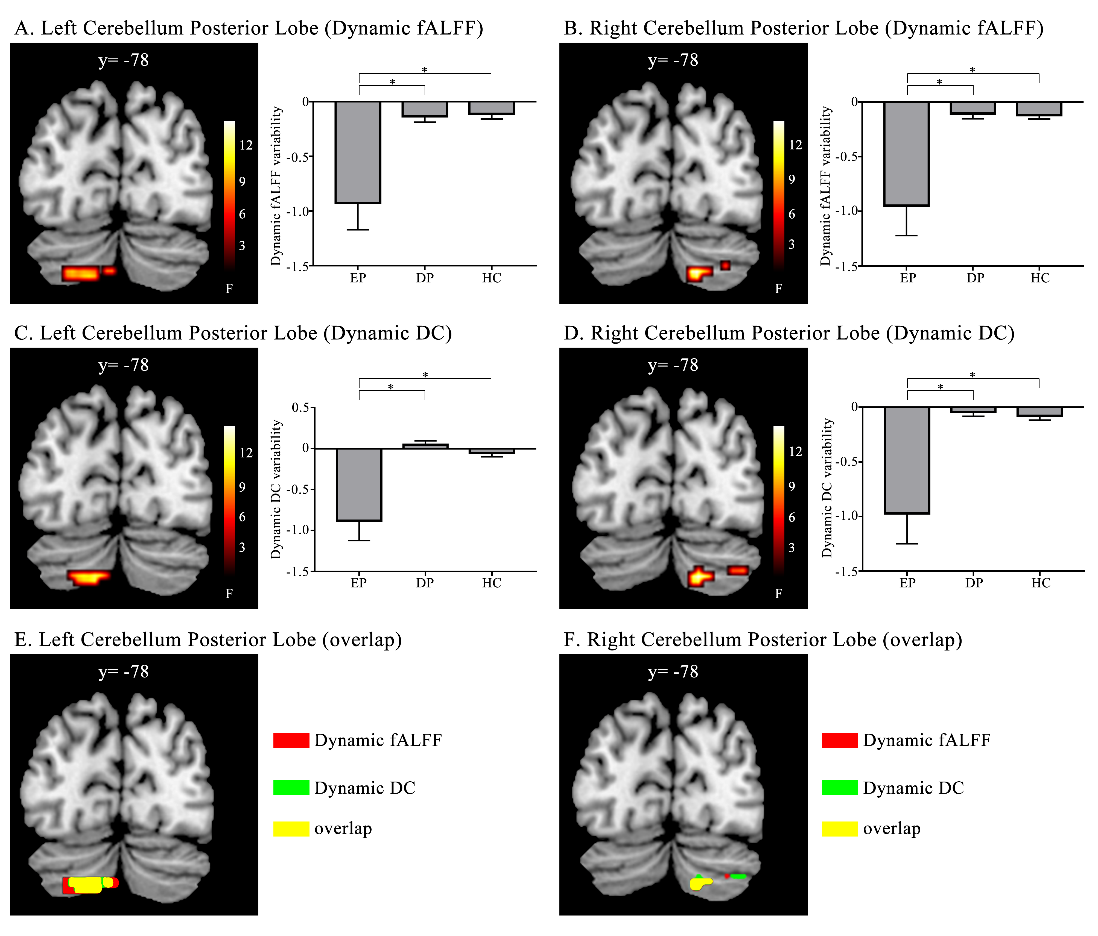


**Figure S2.**


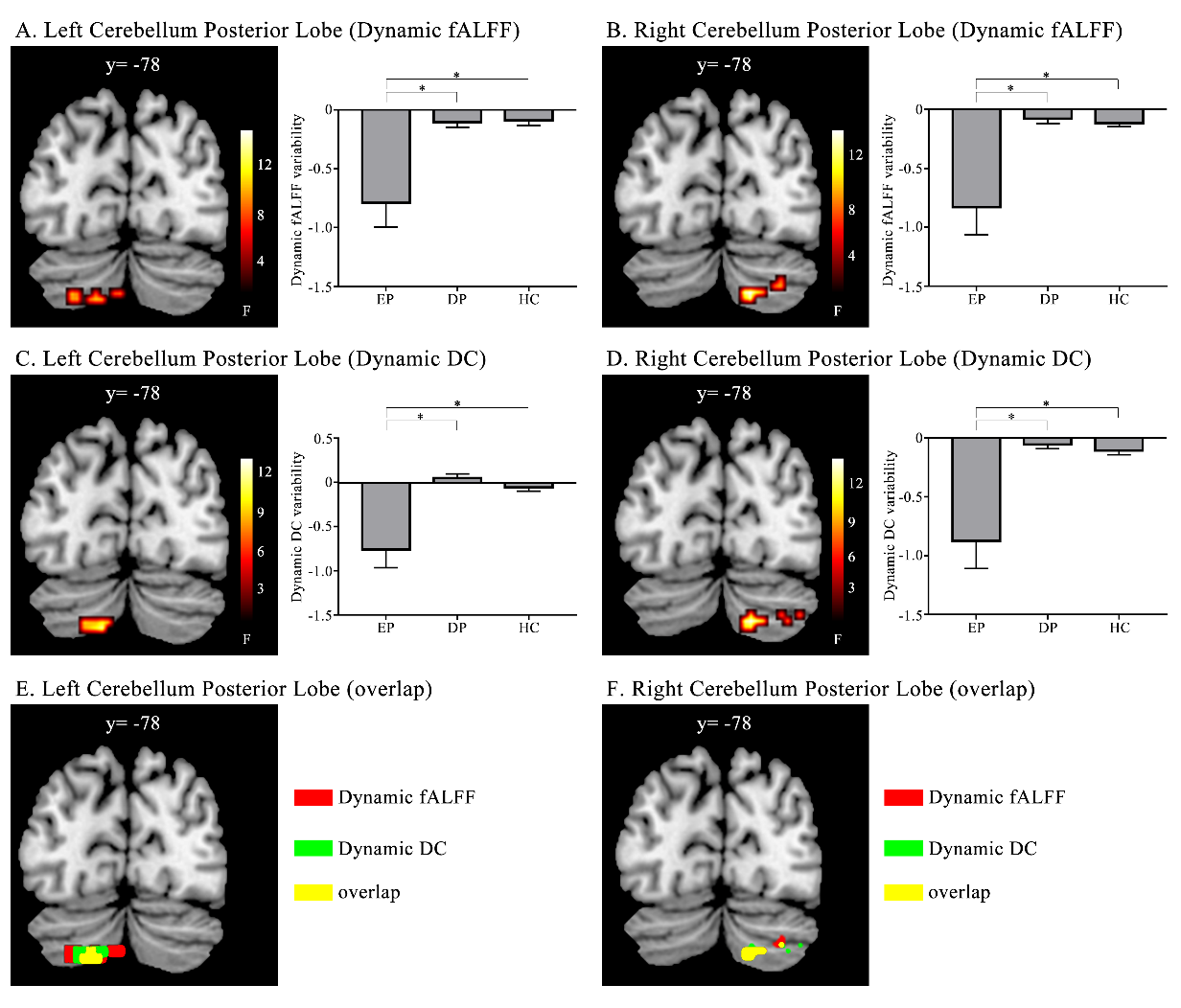


**Figure S3.**


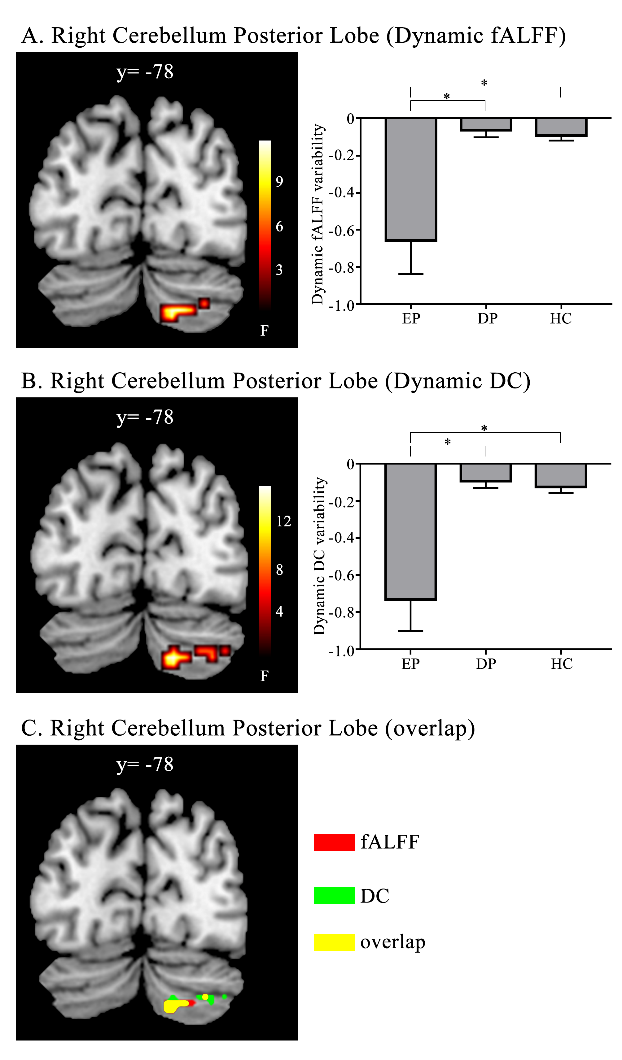


**Figure S4**


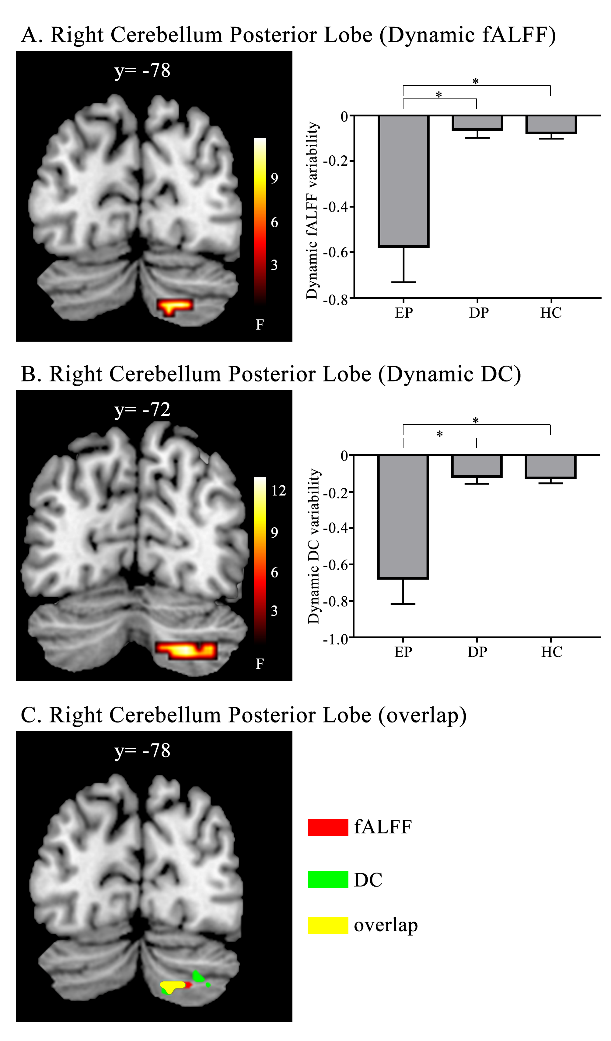


**Figure S5**


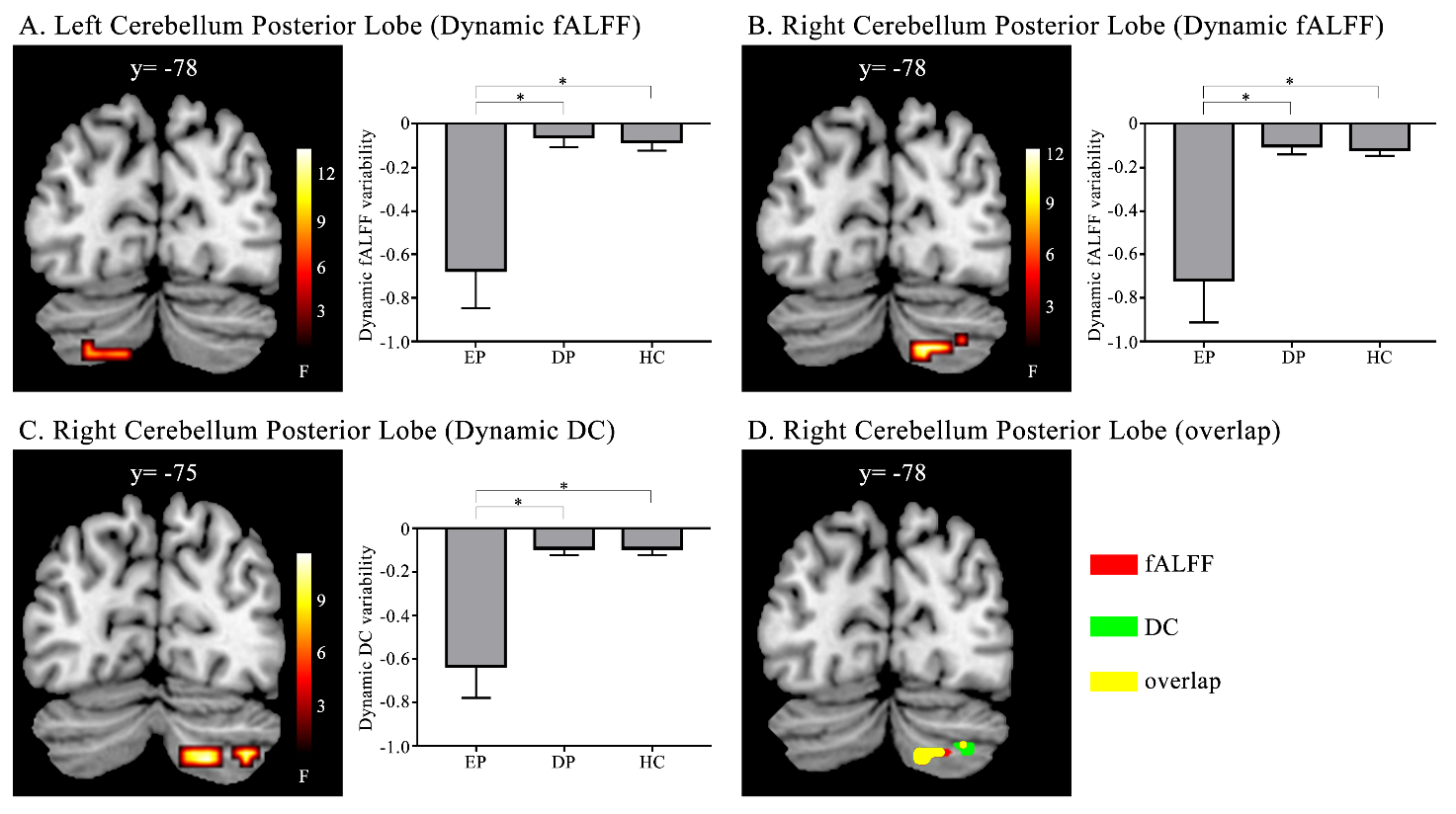


**Figure S6**


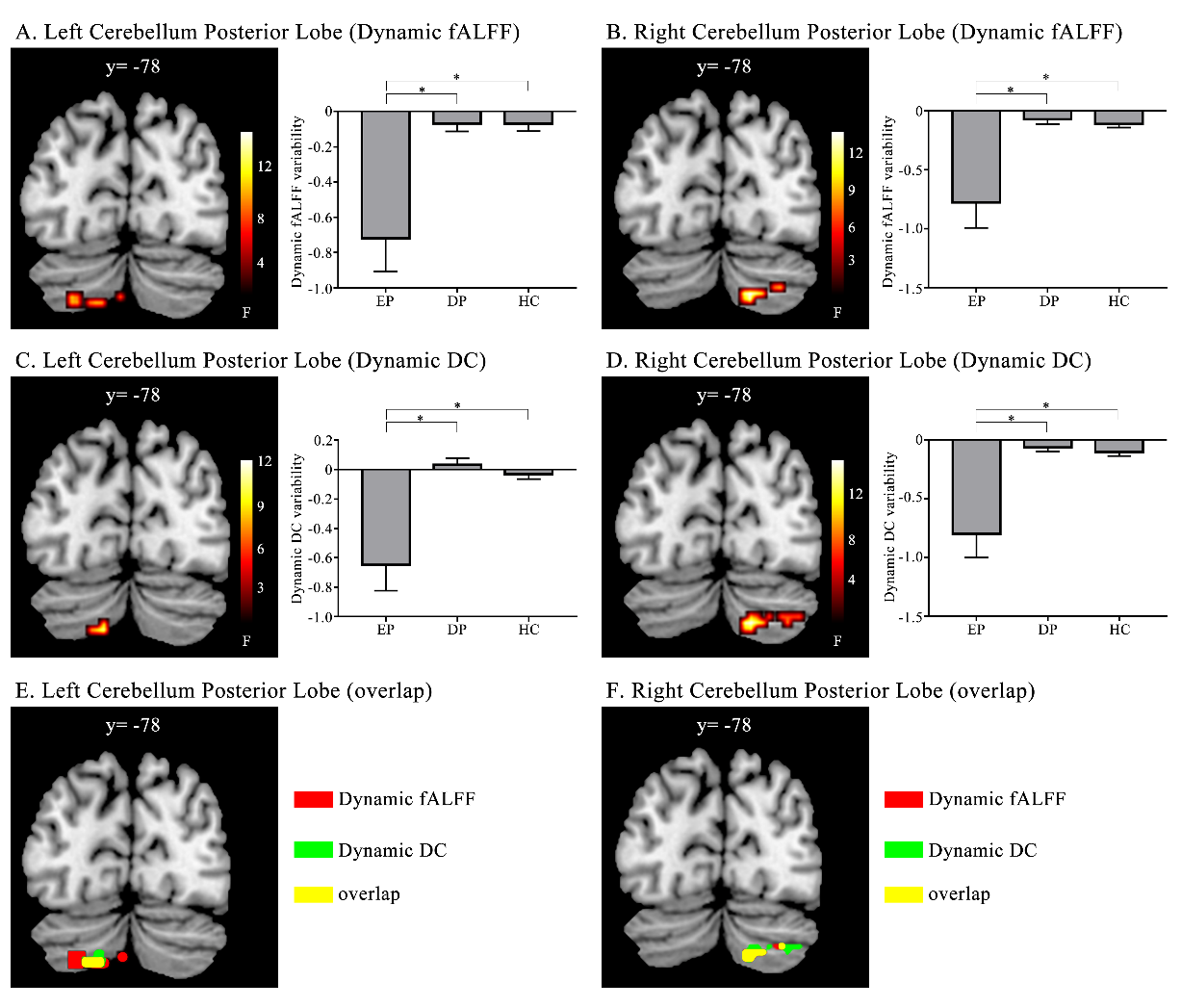

Supplement: Supplementary file 1 — Supplementary Material 1 [file 12888_2024_5596_MOESM1_ESM.doc]
